# Identifying Opportunities to Increase HIV Testing among Mexican Migrants: A Call to Step Up Efforts in Health Care and Detention Settings

**DOI:** 10.1371/journal.pone.0123631

**Published:** 2015-04-10

**Authors:** Ana P. Martínez-Donate, Maria Gudelia Rangel, Natalie Rhoads, Xiao Zhang, Melbourne Hovell, Carlos Magis-Rodriguez, Eduardo González-Fagoaga

**Affiliations:** 1 Department of Population Health Sciences, University of Wisconsin-Madison, Madison, WI, United States of America; 2 El Colegio de la Frontera Norte, Tijuana, Mexico; 3 Graduate School of Public Health, San Diego State University, San Diego, CA, United States of America; 4 Centro de Investigaciones en Infecciones de Transmision Sexual, Programa de VIH y SIDA de la Ciudad de Mexico, la Ciudad de Mexico, Mexico; Universita degli Studi di Roma Tor Vergata, ITALY

## Abstract

HIV testing and counseling is a critical component of HIV prevention efforts and core element of current “treatment as prevention” strategies. Mobility, low education and income, and limited access to health care put Latino migrants at higher risk for HIV and represent barriers for adequate levels of HIV testing in this population. We examined correlates of, and missed opportunities to increase, HIV testing for circular Mexican migrants in the U.S. We used data from a probability-based survey of returning Mexican migrants (N=1161) conducted in the border city of Tijuana, Mexico. We estimated last 12-months rates of HIV testing and the percentage of migrants who received other health care services or were detained in an immigration center, jail, or prison for 30 or more days in the U.S., but were *not* tested for HIV. Twenty-two percent of migrants received HIV testing in the last 12 months. In general, utilization of other health care services or detention for 30 or more days in the U.S. was a significant predictor of last 12-months HIV testing. Despite this association, we found evidence of missed opportunities to promote testing in healthcare and/or correctional or immigration detention centers. About 27.6% of migrants received other health care and/or were detained at least 30 days but not tested for HIV. Health care systems, jails and detention centers play an important role in increasing access to HIV testing among circular migrants, but there is room for improvement. Policies to offer opt-out, confidential HIV testing and counseling to Mexican migrants in these settings on a routine and ethical manner need to be designed and pilot tested. These policies could increase knowledge of HIV status, facilitate engagement in HIV treatment among a highly mobile population, and contribute to decrease incidence of HIV in the host and receiving communities.

## Introduction

Highly mobile populations are more vulnerable to HIV and other infectious diseases and can play an important role in the relocation and spread of disease between home and host countries. In 2013, over 320,000 Mexican migrants traveled to the Mexican border to go to the U.S. and close to 703,000 Mexicans returned to Mexico from the U.S.[1,2] A survey of migrant flows in the Mexico-U.S. border found HIV rates are above those of adults in the U.S. and Mexico.[3] In Mexico, a large proportion of HIV infection has been associated with migration to the U.S.[4–6]

In the U.S., approximately 20% of people infected with HIV are unaware of their infection and this group is estimated to be responsible for nearly half of new transmissions.[7] Undiagnosed HIV infected individuals are more likely to engage in riskier behavior and transmit the infection to others compared to those who are aware of their positive status. The former may not achieve the same quality-of-life adjusted years benefit as those who know they are infected and start treatment earlier.[8] Previous studies have documented barriers to HIV testing for Latino immigrants in the U.S., including undocumented immigrant status, lack of knowledge regarding HIV risk, limited access to health care,[9] and social stigma.[10] Late HIV testing is a prevalent problem for foreign-born Latinos in the U.S., with about 43% of HIV-infected cases progressing to AIDS within a year of diagnosis.[11] In Mexico, it is estimated that 52% of HIV cases are undiagnosed[12] and previous research has documented high levels of late HIV testing (approximately 61% of new HIV cases), which contribute to a lack of reduction in AIDS deaths.[13]

Every year approximately 400,000 Mexican migrants return to their home country from the U.S.[1] and an additional 250,000 are forcedly removed or deported to Mexico.[14] Circular migration, defined as repeated migration experiences between communities of origin and destination involving more than one migration and return,[15] may increase the risk for HIV infection, as well as create barriers for HIV testing and engagement in care. Mexican migrants who repeatedly travel back and forth between sending and receiving communities either voluntarily or by force represent a highly mobile population at risk for HIV infection. A survey of returning Mexican migrants found that 89% of migrants who tested positive for HIV were unaware of their HIV status.[3] Identifying and seizing opportunities to promote HIV testing among circular Mexican migrants could help protect their health, as well as lower HIV incidence and improve rates of engagement into HIV care in both the U.S. and Mexico.

Routine, voluntary, opt-out HIV testing in health care settings is an important strategy for identifying infected individuals and decreasing the spread of the disease[16,17] but the potential of this approach to increase HIV testing among circular Mexican migrants needs to be evaluated. Although Mexican migrants represent a medically underserved population, a substantial proportion interacts with the U.S. health care system. A recent survey of Mexican migrants returning from the U.S. estimated that 42% of them received healthcare services during the previous 12 months.[9] The Centers for Disease Control and Prevention (CDC) recommend that health care providers test everyone between the ages of 13 and 64 at least once as part of routine health care.[16] A recent study estimated that optimal, cost-effective HIV testing would be testing every 2.4 years for low-risk individuals, every 9 months for individuals at moderate risk and every 3 months for high-risk individuals.[18] Given their mobility, poor socioeconomic status, and limited access to health care, migrants should be considered a moderate to high-risk group and be routinely offered HIV testing by health care providers. However, the extent to which Mexican migrants accessing health care services are being offered and receiving HIV testing in health care settings in the U.S. is unknown.

Prisons, jails, and immigration detention centers also offer a unique opportunity to provide prevention services to individuals at high risk for disease who may not otherwise interact with the health care system or be identified and treated in the general community.[19] Mainly because of their unauthorized immigration status, a large number of Mexican migrants are arrested and detained in immigration centers, jails, or prisons and ultimately deported back to Mexico every year.[20] According to Mexico’s National Institute of Migration, 330,000 migrants were deported from the U.S. to Mexico in the year 2013 alone.[21] Data from a survey on the Mexico-U.S. border indicate that over a third of Mexican migrants returning to Mexico voluntarily or via deportation have been detained in a detention center or prison in the U.S. during the last 12 months.[3] These numbers suggest opportunities to promote HIV testing among Mexican migrants in health care settings, correctional systems, and detention centers. The National Commission on Correctional Health Care (NCCHC)[19](National Commission on Correctional Health Care 2002)(National Commission on Correctional Health Care)[19] recommends that correctional systems incorporate easy, convenient, and voluntary HIV testing into the intake procedure for all inmates not known to have HIV.[19] The CDC also recommends HIV testing upon entry into prisons and before release, with voluntary testing offered periodically during incarceration.[22] In practice, HIV testing in correctional facilities varies by state and more than 50% of state prisons do not require HIV testing at any point, although testing is reportedly available to prisoners who request it.[23] Similarly, U.S. Immigration and Customs Enforcement (ICE) standards require detainees to receive a medical screening within 12 hours of arrival at a facility and a comprehensive health assessment within 14 days.[24] However, this assessment does not necessarily include the provision of voluntary HIV counseling and testing. A detainee may request an HIV test at anytime, but testing is not routinely offered or encouraged.[25] International guidelines also establish that prisoners should have a right to health care that is equivalent to the surrounding community, including access to HIV voluntary testing, means of prevention, treatment and confidentiality.[26,27] Little research has examined HIV testing of immigrants in correctional or ICE detention centers in the U.S. Since ICE does not have a mandate to provide statistics on HIV, limited data are available on HIV testing and medical care for immigrant detainees. However, limited evidence suggests inadequate initial screening for HIV and lack of effective efforts to maintain a confidential, free from harassment environment for HIV-positive detainees.[28] In this study, we estimate last 12-months rates of HIV testing among circular, mostly undocumented Mexican migrants returning from the U.S. to Mexico voluntarily or via deportation. We also analyze the role of health care, correctional, or ICE detention systems in promoting HIV testing and missed opportunities to increase testing rates in these settings among this vulnerable and medically underserved population.

## Methods

From April to December 2013, we conducted a cross-sectional, probability health care survey (N = 1161) of mostly Mexican migrants that were returning from the U.S. either voluntarily (a.k.a Southbound flow, N = 695) or via deportation (a.k.a. Deported flow, N = 466). The survey was conducted in Tijuana, Mexico. In 2013, the Tijuana-San Diego border region concentrated about 30% of the Southbound migration flow between Mexico and the U.S.[2] and the highest number of repatriation events in the U.S.-Mexico border, accounting for 14% of them.[21] Samples size was calculated for the main objective of the survey, namely to estimate with relative precision levels of health care access and utilization among Mexican migrants. Estimates were obtained based on a previous pilot health care survey conducted in Tijuana with the same population.[9] Once the overall sample size was determined, the target sample sizes for each migrant flow were calculated to be proportional to the volume of each migration flow observed on previous survey waves.[3]

The sampling methodology was designed after the Mexican government-funded Migration Survey on the North Border of Mexico (EMIF, its Spanish acronym, www.colef.mx/emif). Survey venues included the largest bus station, airport and deportation facility in Tijuana. Survey time units included the day of the week and survey shit. Every quarter a random sample of “venue-time” pairs was selected to determine where and when the survey was to be conducted over the following 3 months. The selection of sites and time units was done proportionally to the volume of the migrant flow traveling through each venue and time period.

During each survey shift, individuals crossing through the sampling points were consecutively approached and screened for eligibility by a Mexico-native, trained research assistant. Eligible individuals were defined as those 18 years or older, born in Mexico or other Latin American countries, fluent in Spanish, not a resident of Tijuana (except for deported migrants), and not have participated in the survey before. An interviewer-administered, computer-assisted questionnaire was used to collect information on last 12-month HIV testing, socio-demographics (i.e. age, gender, marital status, education, and health insurance status), migration history (i.e. time spent in the U.S. overall and in the last 12 months, migration trips, illegal crossing, and intentions to return to the U.S. within the next year), and contextual factors from eligible, consenting individuals. Additional questions assessed whether respondents had received other health care services (different from HIV testing) or had been detained in an immigration or correctional setting for at least 30 days in the U.S. during the 12 months prior to the survey. Oral informed consent was obtained from each survey participant to reduce the risk of exposing personal identification. The oral consent was documented both by the interviewers and in the electronic questionnaire. All study procedures were approved by the Social and Behavioral Sciences Institutional Review Board at University of Wisconsin, Madison, and Ethics Committee of the Mexico-U.S. Border Health Commission. The sampling strategy, eligibility criteria, recruitment, and data collection procedures have been described in more detail elsewhere.[29]

We calculated descriptive statistics of the rates of HIV testing, other healthcare receipt, and detention/imprisonment during the past 12 months. Logistic regression models were run to identify the association of last 12-months receipt of other health care services and incarceration or detention for at least 30 days in the U.S. with HIV testing. We estimated a model with all migrants included and two separate models for each migration flow (southbound vis a vis deported). Models included sociodemographics (gender, age, education, and marital status). The overall model further included migration flow (southbound or deported) and two interaction terms between migration flow and healthcare receipt and migration flow and incarceration or detention as control variables. Survey weights were computed and used to account for the complex sampling design and refusals. Survey weighting procedures have been described elsewhere.[30] Consistent with previous recommendations, survey weights were used to produce population-level descriptive estimates, while regression models were estimated with unweighted data to obtain smaller standard errors and produce more efficient parameter estimates.[31] All analyses were performed with the software STATA/MP 13.1 (StataCorp LP, College Station, TX).

## Results

Among 2,372 eligible individuals, 1,161 (695 southbound and 466 deported migrants) agreed to participate in the survey and provided complete data for this study, yielding a final response rate of 67.3% (55.8% for the Southbound and 97.0% for the Deported). Table 1 shows that overall Mexican migrants were generally young (mean age = 43.1 years, SD = 13.9), primarily male (77.1%), and low educated (only 33.3% completed high school). On average, migrants had spent 17.2 years (SD = 12.4) in the U.S. and had migrated to the U.S. three times in their lifetime to work or to find a job (Median = 1, Interquartile range = 1–2). Approximately, 68.2% crossed the border illegally last time and 74.4% of migrants intended to return to the U.S. within the next year. During the precedent year, only 43.6% had health insurance in the U.S.

**Table 1:** Socio-demographic, migration characteristics of Mexican migrants returning from the U.S. via Tijuana, Mexico.

	Overall (Sample N = 1,161; Weighted N = 227,888)	Southbound (Sample N = 695; Weighted N = 194,343)	Deported (Sample N = 466; Weighted N = 33,544)	P*
Male, *%*	77.1	75.1	88.1	<.001
Age, *Mean (SD)*	43.1 (13.9)	45.1 (14.1)	34.8 (10.2)	<.001
Completed high school education, *%*	33.3	35.6	20.2	<.001
Married/cohabiting, *%*	59.8	61.7	48.4	.001
Time spent in the US during lifetime (years), *Mean (SD)*	17.2 (12.4)	17.7 (10.9)	13.8 (13.9)	<.001
Time spent in the U.S. last 12 months (days), *Mean (SD)*	263.0 (126.0)	264.7 (102.4)	253.1 (235.9)	.289
Migration trips to the U.S. during lifetime, *Mean (SD)*	3.2 (8.0)	3.3 (7.1)	3.1 (7.6)	.734
Entered the U.S. legally last time, *%*	68.2	78.7	8.6	<.001
Intends to return to the U.S. within next year, *%*	74.4	80.8	35.9	<.001
Having any health insurance during last 12 month in the U.S., *%*	43.6	47.2	21.1	<.001
Incarcerated/detained more than 30 days in the U.S. during last 12 months, *%*	9.3	3.6	42.1	<.001
Received any type of health care in the U.S. during the last 12 months, *%*	42.2	43.1	37.1	<.001
Tested for HIV during the last 12 months, *%*	22.0	21.3	26.3	0.184

* Probability values are based on Chi square tests (binary variables) and t tests for independent samples (continuous variables) comparing estimates for southbound migrants versus deported migrants.

In general, deported migrants were younger, more likely to be male, but less likely to be married or cohabiting with a partner compared to southbound migrants (p<.001). On average, the former had spent less time in the U.S. during their lifetime (p<.001). In addition, they were less likely to report a legal last entry into the U.S., intentions to return to the U.S. within the next year, having health insurance, and receiving other health care services during the last 12 months in the U.S. than their southbound counterparts. Deported migrants were more likely to report having been incarcerated or detained for 30 days or more compared to southbound migrants (p<.001).

**Fig 1:**
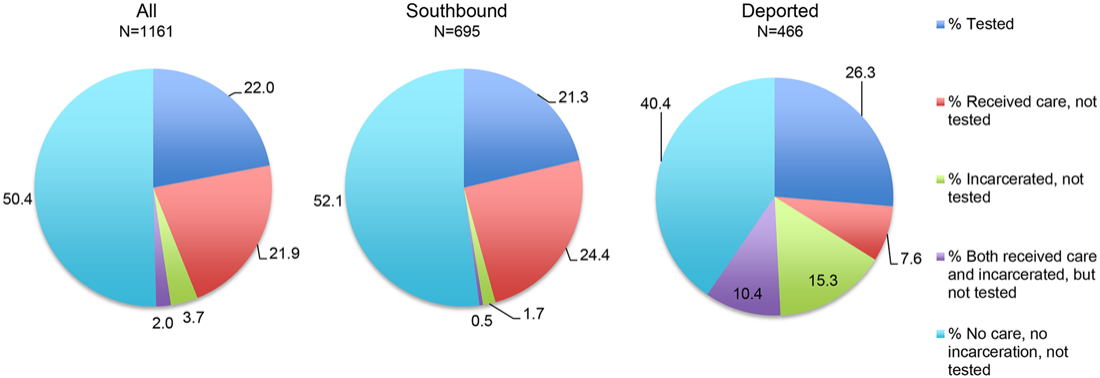
Last 12-month HIV testing, healthcare receipt and incarceration in the U.S. among Mexican Migrants.

Approximately, 22.0% of all migrants (21.3% of southbound migrants and 26.3% of deportees) received HIV testing in the last 12 months (Fig 1).

We found that 23.9% of migrants received other health care services in the prior 12 months (24.9% of southbound and 18% of deported migrants), but were *not* tested for HIV infection. Likewise, 5.7% of all migrants were detained in jail or a detention center for at least 30 days, but *not* tested for HIV during the last 12 months. Compared to southbound migrants (2.2%), a higher proportion of deportees (25.8%) were detained but not tested for HIV (p<0.001). When combining the two settings, the percentage of migrants who had received other care services and/or been detained but *not* tested was 27.6% (26.6% of southbound migrants and 33.3% of deported migrants). These represented over a third (35.4%) of all non-tested migrants (33.8% of non-tested southbound and 45% of non-tested deported migrants; Fig 1).

Regression models indicated that receipt of other health care services (OR = 10.6, 95% CI = 7.38, 15.1) and incarceration or detention for at least 30 days (OR = 1.79, 95% CI = 1.14, 2.80) increased the likelihood of last 12-month HIV testing, after controlling for migration flow (for the model estimated with the overall sample), gender, age, education, and marital status (Table 2). The odds ratios estimated for interaction terms of migration flow*health care receipt and migration flow*incarceration included in our overall regression model were not statistically significant, suggesting that the associations between receipt of health care, incarceration, and the likelihood of HIV testing were not significantly different for southbound and deported migrants.

**Table 2:** Association between receipt of health care services and incarceration or detention with the likelihood of reporting last 12-months HIV testing among circular Mexican migrants returning from the U.S. via Tijuana, Mexico^1^.

	Overall^2^ (Sample N = 1,161) AOR (95% CI)	Southbound (Sample N = 695) AOR (95% CI)	Deported (Sample N = 466) AOR (95% CI)
Received other type of health care in the U.S. during last 12 months	**13.1 (7.80–22.0)** **	**13.9 (8.21–23.5)** **	**8.89 (5.26–15.0)** **
Incarcerated/detained for 30 or more days in the U.S. during last 12 months	**2.8 (1.13–7.01)** *	**2.71 (1.07–6.85)** *	**1.70 (1.01–2.87)** *

^1^ Based on logistic regression models that included last 12-months HIV testing as the outcome and receipt of other health care services and detention for 30 or more days as the main predictors. Models were estimated for the whole sample and for each migration flow separate. Migration flow (for overall sample only), gender, age, education, and marital status were included in the models as covariates.

^2^ The overall model was further adjusted by migration flow and included two interaction terms: migration flow*receipt of care and migration flow*incarceration. The odds ratios estimated for these interaction terms were not statistically significant.

*p<0.05,

**p<0.01.

## Discussion

Our study shows that four out of five migrants have not been tested for HIV in the last 12 months. The rates are similar to those found for adults 18–64 in the U.S.[32] However, considering previous research showing high rates of HIV infection, behavioral risk factors, and late HIV diagnosis among Mexican migrants,[33–36] these results suggest the need to amplify existing efforts, and develop new strategies, to more effectively promote HIV testing among this hard-to-reach, transnational risk population.

Our models indicate that interactions with health services, immigration, or correctional systems were the strongest predictors of HIV testing. The odds of having received an HIV test were almost 11 and 2 times more likely among migrants who had received *other* health care services or had been incarcerated or detained for at least 30 days, respectively, compared to their counterparts who had not accessed other health care services or been detained. Previous research has shown that among circular Mexican migrants, access to health services is associated with the availability of health insurance and transportation.[9] Findings from this study further suggest that interventions to promote access to health care services, through initiatives to expand health insurance options and reduce transportation barriers, could also contribute to increase HIV testing among this population. Under the Affordable Care Act (ACA), undocumented, circular migrants are still not eligible for insurance coverage, so it will be important to document how the implementation of the ACA impacts access to and sources of health care among this population. Some early reports suggest there may be a reduction of services available to uninsured populations, including migrants, particularly in states that have opted not to expand Medicaid.[37–39] Limited insurance rates may impede linkage to care and continuity of HIV treatment among migrants who are found to be HIV positive, seriously undermining the public health impact of initiatives to expand HIV testing among this population. The association between incarceration/detention and HIV testing suggests some migrants may be accessing HIV testing while imprisoned or detained in immigration facilities. On the other hand, it is also possible that being in prison or an immigration detention center increases future testing outside these settings, through increased perception of risk among migrants with these histories.

In general, our results suggest that windows of opportunity in health care and correctional/immigration detention settings are *to some extent* being utilized to provide HIV prevention services to a hard-to-reach, at-risk population. However, the results also show substantial room for improvement. Over 1 in 4 (27.6%) migrants returning from the U.S. to Mexico have received health care services in the U.S., been held in a detention center or prison in the U.S. for a minimum of 30 days, or experienced both in the past 12 months, but have *not* been tested for HIV. These findings are consistent with other studies, suggesting missed opportunities for testing for highly vulnerable populations in healthcare[40,41] and correctional settings.[42–44] Our data suggest that health care settings, correctional, and immigration detention centers in the U.S. could be more effectively harnessed to step up HIV testing efforts and reduce the number of migrants who are unaware of their HIV status, especially among deported migrants. They also call for additional mixed-methods research to understand the reasons behind these missed opportunities and effective interventions to promote HIV testing in these settings.

In contrast with CDC recommendations for routine, opt-out HIV testing of adolescents and adults in health care settings, our findings indicate migrants are not consistently being tested for HIV when they access other health care services in the U.S. Low health insurance rates,[9,45] insufficient training in HIV testing and treatment,[46,47] and limited cultural and linguistic competence,[45,48] among other factors, may prevent health care providers from routinely offering HIV counseling and testing to Mexican migrants in the U.S. Likewise, HIV stigma, fear of deportation, and low perception of HIV risk may discourage migrants from accepting HIV testing in health care settings.[45,49,50] Future research needs to estimate the extent to which migrants are offered HIV testing when seeking other health care services and identify system, provider, and individual factors associated with offering and acceptance of HIV testing in health care settings for this population. Research should also differentiate between primary, specialty, and emergency health settings, as rates and opportunities for HIV testing in these contexts may vary substantially.[51]

Our data suggest that recommendations and human rights standards that call for voluntary HIV testing and adequate prevention and care for individuals held in detention centers and prisons are not standard protocol. The findings are consistent with previous reports concluding that immigrant detention and correctional settings fail to regularly screen detainees for HIV.[28,52,53] The correctional system’s lack of clinical guidelines for prevention, screening, and treatment of HIV has been noted as a critical limitation for the prevention of the spread of HIV infection in these settings.[19] A 2008 report found that a total of 24 states reported testing all inmates for HIV at admission or sometime during custody. Among these 24 states, 23 tested at admission, five tested while in custody, and six tested upon release. Fifty states and the federal system tested inmates if they had HIV-related symptoms or if they requested an HIV test. Forty-two states and the federal system tested inmates after they were involved in an incident in which an inmate was exposed to a possible HIV transmission, and 18 states and the federal system tested inmates who belonged to specific high-risk groups.[54] Reviews of detainees’ health in ICE facilities also found that HIV testing policies and procedures were conflicting and incomplete, causing confusion and lack of standardization.[52,53]

Missed HIV testing opportunities in health care, correctional, and immigration detention settings have important public health implications for both the U.S. and Mexico. Given the transnational nature of circular Mexican migrants and the likely benefits of lowering migrants’ HIV risk for the U.S. and Mexico, it would be in the best interest of the two countries to collaborate in the design, financing, implementation, and evaluation of interventions to increase HIV testing among this population in U.S. health care, correctional, and immigration detention settings. However, before issuing recommendations to roll out and enforce HIV testing policies, the impact of routine, voluntary, and confidential HIV prevention, screening, and treatment of migrants in these settings needs to be evaluated. Based on our probability-based sample, we speculate that if non-tested migrants who received other healthcare services and/or were detained for at least 30 days had been encouraged, and agreed to be counseled and tested for HIV in these settings, the overall rate of HIV testing in this population could have increased to 50%, a 125% increase over current testing rates. Past evidence indicates that when offered, acceptance rates of HIV testing tend to be high among migrants.[3,55] A survey of 700 deported migrants conducted at a deportation station in Tijuana, Mexico, showed that 80% of them agreed to be tested for HIV,[33] suggesting acceptance can be high when certain conditions (e.g. informed consent, voluntary participation, anonymous, rapid, non-invasive methods, trained, culturally and linguistically congruent staff, etc.) are met.

Counseling and testing for HIV has been found to be a cost-effective and cost-saving intervention in U.S. prisons. Some estimates indicate that for every 10,000 inmates being offered HIV counseling and testing, if 60% of them agreed to be counseled and tested, three cases of HIV infection could be prevented, resulting in savings of $410,000. Even with prevalence rates as low as 1.5%, the intervention would be cost effective in correctional settings.[19] While it is not known if these practices would be cost effective in ICE detention centers, surveys have found rates of HIV infection among different migrant flows traveling through the Mexico-U.S. border ranging from. 08% to 3.89%.[3] The use of more advanced HIV testing technology should also be evaluated for its cost-effectiveness in these settings. Fourth generation testing assays can identify individuals with acute HIV-1 infection, when an individual is most contagious. All other testing technologies have window periods exceeding the acute phase, which can result in false negatives.[56] Fourth generation testing assays have higher specificity and sensitivity, which may be especially appropriate for migrant populations that are transient in nature and have higher rates of HIV. However, certain limitations still exist such as additional costs, more waiting time for the result, and the need for confirmatory testing for a positive result. Research formally evaluating the effects on routine, opt out HIV testing in ICE detention centers on migrants’ testing rates, undiagnosed HIV cases, engagement and retention into care, and prevention of HIV transmission to others must be conducted and used to inform future policies and guidelines for HIV testing of migrant populations in health care, correctional, and immigration systems.

The case of deported migrants deserves special consideration. Our data show that the percentage of deported migrants who could have been reached in health centers, prisons, or ICE detention centers is even greater than among other subsets of returning migrants (33.3% among deportees versus 26.6% among southbound migrants). After being held by U.S. immigration authorities, most deported migrants are transferred to deportation stations located in the Mexican border and run by Mexico’s National Institute of Migration. Deported migrants are then released into the general community of these border towns with no or little support or resources to survive. The U.S. and Mexico have an ethical obligation to ensure that to-be-deported and deported individuals obtain appropriate medical care prior to deportation or release into the general community. ICE detention centers in the U.S. and deportation stations in Mexico offer a critical window of opportunity to offer HIV testing and counseling, prevention education, and linkage to care in the Mexico border. Given the size, mobility, and transnational nature of this population, HIV counseling, testing, and linkage to care prior to and immediately after deportation can not only protect the health of deported migrants, but lower the risk of transmitting the disease to others in sending, receiving, and border communities.

In Mexico, some efforts are already underway to provide prevention and health promotion services, including routine, opt-out HIV testing, to deported migrants in deportation stations located in the border cities of Tijuana and Mexicali. Similar clinics are planned to open in deportation stations based in Matamoros, Nuevo Laredo, and Reynosa. In the first 18 months of operation, the Tijuana clinic provided services to almost 3,000 migrants. About 80% of them agreed to be tested for HIV and, among them, ten cases of HIV infection were identified and linked to HIV treatment services. Unfortunately, limited resources restrict the capacity of this clinic to serve about 3% of all migrants repatriated through these deportation stations (G. Rangel, Mexico-U.S. Border Health Commission, personal written communication, August 6, 2014). Future research must be conducted to evaluate the impact of these programs and, if deemed cost-effective, expand their number and capacity. In any case, these services offer a start that might be coordinated with services on the U.S. border for similar surveillance and triage to care as needed.

### Limitations

This study is subject to several limitations, including a cross-sectional design, moderate response rates, data solely based on self-reports, and Tijuana as the only site. No testing could reflect testing was not offered or made available to migrants in health care or detention settings, but it could also reflect testing was offered *but* not accepted by migrants. Future studies need to be designed so these two scenarios can be differentiated. At any rate, the results suggest opportunities for improving testing rates through interventions that increase testing availability and/or acceptance.

## Conclusion

Mobility, poor living and working conditions, and low levels of health care access place Mexican migrants at risk for HIV infection and other infectious diseases. Our survey reveals limited rates of recent HIV testing among this population and significant variations according to migration history, plans, and interaction with health care, immigration, and correctional systems. A substantial proportion of non-tested migrants could be reached in health care, correctional, ICE detention centers, and deportation stations. These settings offer critical opportunities to intervene and lower the rates of undiagnosed and untreated disease among a vulnerable and medically underserved population. Policies and programs to increase access to health care services and to offer routine, opt-out HIV testing of Mexican migrants in health care settings, prisons, and immigration centers should be pilot tested given their potential to link HIV positive migrants to care, reduce HIV incidence, and improve overall levels of engagement in HIV care in both the U.S. and Mexico.

## Supporting Information

S1 Full Dataset Healthcare Access and Utilization and HIV Testing among Mexican Migrants. (DTA)
